# In-vitro metaphors: ART beneficiaries’ meaning-making about human embryos in the context of IVF in Portugal

**DOI:** 10.1016/j.rbms.2021.05.003

**Published:** 2021-06-29

**Authors:** Catarina Delaunay, Mário J.D.S. Santos, Luís Gouveia

**Affiliations:** a Centro Interdisciplinar de Ciências Sociais, Universidade NOVA de Lisboa, Lisbon, Portugal; b Comprehensive Health Research Centre, Universidade NOVA de Lisboa, Lisbon, Portugal; c NOVA National School of Public Health, Public Health Research Centre, Universidade NOVA de Lisboa, Lison, Portugal; d Instituto Universitário de Lisboa, Lisbon, Portugal

**Keywords:** In-vitro fertilization, Embryo conceptualization, Sociological factors, Qualitative research, Metaphor

## Abstract

This article proposes a metaphorical approach to the meaning-making of Portuguese assisted reproductive technology (ART) beneficiaries about human embryos created *in vitro*, based on the analysis of 30 in-depth interviews. This article draws from an ongoing research project on expert and lay definitions of human embryos developed *in vitro*, both in ART and scientific research. Four metaphors were identified in patients’ utterances about the embryo’s status and attributes: embryos are possibilities of success of treatment; utilities that can be the object of ownership and dispositional control; potential offspring with whom there are family and emotional ties; and a counter-gift to science or to other beneficiaries, in response to the generosity of professionals or gamete donors. These rhetorical devices seem to ease the tensions inherent in the technical procedures of medically assisted reproduction. Examining the meaning of attributive metaphors used by patients undergoing in-vitro fertilization about their embryos *in vitro* is thus essential to understanding their personal experiences, so that healthcare professionals can direct their actions/interventions towards their specific needs and concerns, which are not always spoken.

## Highlights

• In-vitro fertilization (IVF) requires the development of human embryos in a laboratory.
• Beneficiaries of IVF give different meanings to these embryos.
• The interviewees described embryos as possibilities, utilities, offspring or as a counter-gift.
• Embryos have a complex social existence, constructed through different metaphorical meanings.

## Introduction

The expansion of assisted reproductive technology (ART) has been reframing the landscape of reproductive care and the experience of parenthood itself. There is a growing body of knowledge within the social sciences that explores the family and wider social implications of ART, reflecting on how these technologies are, in fact, changing human relations or making way for new bonds and networks. This article explores the meaning-making processes and metaphors used by ART beneficiaries to describe the relationships they establish with (their) human embryos *in vitro*. This article began as a presentation at the conference ‘Making families through assisted reproductive technologies: causes, experiences, and consequences in international context’, organized by Anne-Kristin Kuhnt and Jasmin Passet-Wittig, in September 2019, from which this symposium volume stemmed.

Metaphors inhabit the landscape of medicine, namely the medical language and thinking involved in diagnostic work and patient–doctor communication. Examples include ‘the body as a machine’ – a mechanical perspective on the human body, which may be broken and in need of repair – and ‘medicine as war’ – where diseases are viewed as enemies invading the body, and any therapeutic intervention is a defensive manoeuvre – both of which may conflict with other values or perspectives on health care, such as person-centred care (Bleakley, 2017). Metaphors are prevalent in the way that people conceptualize and talk about their illness. According to Jenny and Logan (1996), metaphors provide vivid and powerful images of patients’ concerns, expectations and needs, offering a way for people to express meaning and feeling. These metaphorical expressions can be very graphic, colourful and even intriguing.

Likewise, a diversity of metaphors is used by both laypeople and experts to speak about infertility – which is considered a disease by the World Health Organization – and its diagnostic or related treatments, namely the use of ART. The infertile body is metaphorically framed as a defective machine (i.e. one that fails to achieve pregnancy as a desirable biological and social state), as an emblem of the self (associated with a felt stigma due to not complying with social expectations), and as property over which one exerts control or that betrays the care invested in it by denying fertility (Greil, 2002). Other metaphors are also deployed, presenting the infertile body as a resource, a container, a project, a disordered mechanism, a challenge to the natural order of things, or a social agent in itself, with self-determination and personality (Mezinska and Mileiko, 2012).

Infertility metaphors can be found in patients’ narratives about their experience, in the discourse of health professionals, in scientific medical books, and even in the media. Research studies on infertility in Western societies have shown how the most conventional verbal metaphors about infertility reflect a shared assumption of the need for patients to be goal-directed and competitive, associated with individualistic notions of personal success, achievement or failure (Refaie et al., 2018).

According to a rhetorical analysis of the evolution of reproductive metaphors, contemporary discourses of infertility can be explained in terms of mixed metaphors combining past, diverse and sometimes competing perspectives – such as organic and mechanical arguments – that are dependent on their historical uses and on the different layers of medicalization (Jensen, 2015, Jensen, 2016). This has consequences concerning individual agency and accountability: at present, involuntarily childless women are at once powerless and responsible for their condition (*ibidem*).

In the media and self-help books, women who undertake fertility treatment are typically framed, in metaphorical terms, as ‘winners’ or ‘losers’. Treatments become a kind of lottery (De Lacey, 2002), a game, or a race against the ‘biological clock’ (Friese et al., 2006). Meanwhile, infertile patients often prefer to conceptualize the entire process and their desire for a baby as a purposeful battle, a goal-oriented journey (Palmer-Wackerly and Krieger, 2015), or a type of ‘work’ that involves the best employment of their bodily and monetary resources (De Lacey, 2002).

Some dominant metaphors, such as game or battle metaphors, can help people with infertility issues to work out their own experiences and share them with others (family, friends, health professionals) by giving a sense of common purpose. In contrast, battle metaphors may impact negatively on patients by focusing on the biomedical perspective instead of the social and affective dimensions (Refaie et al., 2018).

The majority of studies about embryos created *in vitro* have analysed the emerging metaphors in legal or bioethical documents, scientific literature and the media, specifically concerning stem cell research. Examples are diverse, and include the use of organicist metaphors (crystals, fabrics and fields) to analyse the paradigm shift in developmental biology, namely embryology (Haraway, 1976); the legal representations of embryos as pre-persons, commodities and cyborgs (Fox, 2000); the superhero metaphor based on stem cells’ supernatural powers to fight any disease, thus being presented as our ‘only hope’ for relieving suffering (Burns, 2009); and the role of visualization technologies in media reporting of ethical debates around stem cell research, namely the opposing rhetorical strategies that cast embryos as fluorescent frogspawn (a small ball of cells) on the one hand, or a young human being on the other (Williams et al., 2003). In Portugal, the arguments set out in regulatory documents produced by national-level ethics committees on research using human embryos also reveal different statuses and classifications assigned to the cryopreserved embryo: a biological neo-structure, a laboratory artefact, a human being or a person (Alves et al., 2013).

However, the publications that contribute most to understanding the metaphors attached to in-vitro fertilization (IVF) embryos have focused on surplus cryopreserved embryos (de Lacey, 2017, de Lacey, 2007, de Lacey, 2005, Laruelle and Englert, 1995, McMahon et al., 2003, Nachtigall et al., 2005, Provoost et al., 2012, Soderstrom-Antitila et al., 2001, Svanberg et al., 2001). This has led to general neglect of all the other metaphorical categories of embryos, referring to those who become a take-home baby, or those lost during the therapeutic trajectory (due to early-stage cleavage anomalies, implantation failures, early miscarriages, etc.).

This article analyses the meaning-making of Portuguese ART beneficiaries about human embryos created *in vitro*, based on the metaphorical expressions – and the underlying conceptual mappings – captured in semi-structured interviews about the experiences of these beneficiaries with IVF. This article considers the many different forms, potentialities and outcomes of in-vitro embryos: either those who are implanted successfully and come to term, or those who are miscarried or otherwise lost in the process. By extending the discussion beyond the space of surplus cryopreserved embryos and of those who become live-born children, this article aims to fill a much-needed gap in the literature in thinking about ART and the role of metaphors in the ways that IVF beneficiaries come to understand their relationships with their embryos.

### Setting

Not all countries have the same guidelines, laws and individual policies regarding embryo or gamete donation, the number of embryos allowed for transfer, embryo cryopreservation (e.g. duration of storage) and embryo experimentation/research; in some countries, these matters are not even regulated (IFFS, 2019).

In the case of Portugal, a therapeutic approach to ART prevailed until 2016. It was necessary to comply with both medical and social conditions: a couple had to be in a stable, heterosexual, marital union, and have health problems (either infertility or the risk of transmitting a genetic condition) in order to receive these treatments (Law 32/2006). However, Law 17/2016 gave all women access to ART, regardless of whether they had an infertility diagnosis, their marital status or sexual orientation. There are both private and public ART centres – the latter with significantly longer waiting lists – and women can receive publicly-funded treatments that are free of charge (excluding medication and travel), including IVF and intracytoplasmic sperm injection (ICSI), up to 40 years of age. The Portuguese law and the Portuguese Society of Reproductive Medicine recommend that no more than three embryos should be transferred simultaneously, according to the beneficiary’s consent and to clinical criteria such as age and the number of previous unsuccessful treatments. All viable embryos that are not transferred are cryopreserved for a maximum of 3 years, and this can be extended by 3 additional years at the couple’s request. Depending on what beneficiaries choose, cryopreserved embryos may be used later, donated to other beneficiaries or donated for scientific research. Despite the deliberate creation of embryos for research purposes being prohibited in Portugal, it is lawful to use embryos for biomedical investigation if they are surplus, if they have severe genetic anomalies, or if they have any condition that does not allow their transfer or cryopreservation. In the absence of any of these choices, the embryos are thawed and eliminated after the period provided for in the Law.

The donation of embryos for scientific research is dependent on an express, informed and conscious consent from the beneficiaries for whom they were intended. On the other hand, the effective use of embryos for investigation depends on the authorization of the regulatory body of ART in Portugal – the National Council for Medically Assisted Procreation – which is responsible for assessing the plausibility of the expected result of each research study in terms of its potential ‘benefit for humanity’. This is the case of intended objectives such as the prevention, diagnosis or therapy of embryos; the improvement of ART techniques; and the establishment of stem cell banks for transplantation programmes or any other therapeutic purposes (Law 36/2006).

Analysing Portugal’s regulation within the current legal status of reproductive policy and practice at a global scale (IFFS, 2019), research involving donated unused pre-implantation embryos is currently allowed, with specific approval, in 26 of 36 (72%) countries, and for stem cell research in 28 of 37 (76%) countries. Research involving donated unused pre-implantation embryos is underway in 24 of 65 (37%) countries, and for stem cell research in 21 of 63 countries (33%) (*ibidem*). In terms of the frequency of experimentation performed, research on donated unused pre-implantation embryos is ‘commonly performed’ in two of 66 (3%) countries, ‘infrequently performed’ in 12 (18%) countries, ‘never performed’ in 36 (55%) countries and ‘unknown’ in 16 (24%) countries (*ibidem*). Thus, the present legal framework places Portugal in a minority group of countries where the use of embryos for investigation, with specific approval, is legal. Notwithstanding, the effective use of embryos in scientific research remains an exiguous phenomenon: to date, only one research project has been approved in Portugal (in 2016).

Within all these decision-making processes, patients perform both emotional and cognitive work in relation to their IVF embryos, which includes making sense of their own experience, managing deep emotions, dealing with doubts, enduring heavy responsibilities, and coping with mixed feelings. By examining the meaning of attributive metaphors used by these patients, our analysis sheds light on these complex sets of relations that patients establish with their embryos created *in vitro*. This may support current and future initiatives towards personalized care, enabling healthcare professionals to direct their interventions towards patients’ specific needs and concerns which, for the most part, are not openly expressed in clinical settings.

## Materials and methods

This article is part of a wider, ongoing research project focused on the plural meaning-making processes of experts and laypeople around human embryos *in vitro* – both in the context of ART and clinical research – grounded in a mixed-methods approach. So far, in this project, 30 in-depth, semi-structured interviews have been conducted with ART users, along with 85 responses to an online survey of IVF beneficiaries and a small number of ethnographic observations. The present discussion is focused on qualitative data from the semi-structured interviews that took place between 5 September 2019 and 15 June 2020. Interviewees were ART beneficiaries recruited through social media and informal contacts, varying in gender, marital status (single or in a partnership, whether marital or non-marital), sexual orientation (heterosexual, homosexual or bisexual) and clinical trajectory (namely with or without a medical diagnosis of infertility). There was a preponderance of female respondents (90%), and only two interviewees had formed their parental project within a same-sex relationship. Ages ranged from 32 to 47 years (with an average of 40 years). Except for one Dutch woman, all respondents were Portuguese. The vast majority (*n* = 25) of respondents lived with a partner, whether married or not; and most (*n* = 25) respondents had higher education qualifications, including eight with postgraduate level education. All interviewees were employed.

All interviews were conducted in person by the same member of the research team. All ethical requirements were met, and informed consent was sought prior to each interview. Besides sociodemographic variables, the interview guide to IVF beneficiaries included questions grouped under the following umbrella topics: parental project; infertility diagnosis and use of ART; knowledge about ART; lived experience of the clinical protocol/trajectory; decision on surplus embryos; and general conceptions about the embryos. All data were anonymized, and all references to specific persons and institutions were eliminated.

Concerning the plurality of clinical courses and their possible impacts on the meaning-making processes for embryos, each interview excerpt presented in this article is accompanied by a matrix of acronyms to describe the participants’ therapeutic trajectory (at the date of the interview). The acronyms refer to five variables and their respective modalities: (i) type of treatment [IVF, ICSI or artificial insemination (AI)] and the number of concluded treatment cycles; (ii) number of obtained embryos (OE); (iii) number of successful treatments (ST; i.e. full-term pregnancies obtained during ART treatment); (iv) number of cryopreserved embryos (CE); and (v) number of surplus embryos discarded (DE). For example, ‘2IVF; 4OE; 1ST; 0CE’ means a clinical course consisting of two completed IVF cycles, with a total of four embryos generated, one pregnancy obtained and zero existing cryopreserved embryos at the date of the interview.

Thematic content analysis of the interviews – which were recorded and transcribed verbatim – was supported by the computer-assisted qualitative data analysis software MaxQDA (2018 version). Data from the interviews and the field notes were analysed inductively using the grounded theory approach (Strauss and Corbin, 1998). The themes analysed in this paper draw primarily from coded segments on the conceptualizations of embryos. Different significant themes were identified, and subsequently coded and categorized, each titled with a name that appeared to capture its implied meanings. After the categories were identified, their emerging sub-themes were analysed and interpreted jointly by the three researchers in order to reach agreement regarding their implicit meaning. Implicit and less conscious verbal metaphors used by the interviewees also carried analytical significance. As such, even in the absence of explicit conceptual metaphors, occasionally data allowed for the interpretation of analogies used by patients undergoing IVF to describe their experience with embryos, which also revealed their underlying metaphorical meanings.

## Results

We identified a grammar of different categories applied to embryos that are deployed by patients undergoing IVF in their discourse about ART treatments. These different narratives about embryos reveal four main metaphors that contribute to the analysis of both the ontological status of embryos and the relationship that IVF beneficiaries establish with them: embryos as possibilities; embryos as utilities; embryos as offspring; and embryos as counter-gifts.

As possibilities, an embryo may represent a new opportunity, in material and functional terms, to try to conceive a biological child as part of a parental project, and therefore it may be perceived as ‘reproductive material’ and a ‘stage of the process’ within ART treatment in order to achieve a pregnancy.

Embryos as utilities refers to their conception as ‘biological matter’, as a ‘valuable resource’ and, therefore, they may be understood as material waste that can easily be discarded when the future expectations placed on them do not involve any additional parental project. Some patients refer to an embryo as a ‘hybrid object in a liminal or suspended state’ that is difficult to seize or define, contrasting with narratives where embryos are perceived as a sprout of human life, triggering feelings of care, love, loss and/or relinquishment.

In contrast with these two pragmatic conceptions, the discourses of some patients undergoing IVF on embryos as offspring convey their perception that a given embryo *in vitro* may become and ‘embody their baby in the future’, and is thus a ‘sibling of their living child’ already born from an IVF embryo. This correlation between an in-vitro embryo and a born child may be even stronger when they come from the same IVF batch. The embryo can also be referred to as a symbol of the couple’s connection, when it seems to materialize the very union between the spouses, either emotionally or genetically speaking.

Finally, in a context of disengagement from the latter biological and affective bond, there are also references to a gift transaction, where embryos detached from the original parental project are described as a counter-gift, as one way to express gratitude and repay reproductive medicine for making parenthood possible. These distinct lines of discourse are linked to the clinical worth of IVF embryos, referring to their moral, social and ontological value, or their contribution to a larger mission and collective endeavour, such as potential advances in scientific knowledge and clinical research.

In order to give a simplified overview of the four prevailing metaphorical categories while also providing some preview of the upcoming discussion of each metaphor, the information has been summarized in Table 1.

**Table 1:** Metaphors for in-vitro fertilization embryos.

Embryo metaphor	Embryo categories
Possibilities	Opportunity for pregnancy Stage of the process Reproductive material

Utilities	Biological matter Valuable resource Hybrid object/liminal entity

Offspring	Sibling of a living child Embodying a future baby Symbol of the couple

Counter-gift	Clinical worth Gift transaction Gratitude expression

### Embryos as possibilities

Some of the interviewees expressed a pragmatic approach to embryos created *in vitro*. For them, embryos are just part of the medical procedure, a means to an end (i.e. the woman successfully getting pregnant). For that reason, embryos are metaphorically perceived and evaluated, by both health professionals and ART beneficiaries, in terms of their quality and aesthetics (sometimes associated with beauty or health), as well as their potential for development and implantation. The ‘weaker or anomalous/defective’ embryos are thus discarded. Their elimination does not trigger any negative feelings of relinquishment or loss, and they vanish from the discourse of both professionals and patients:

Laura: I’m going to be very honest: when we do the treatments, they… they say ‘Look! We got × eggs’. When we did the… the… the transfer, the day we did, they said ‘Look! We are going to put in two and we managed to freeze one’. My head didn’t think of the others. Didn’t think. I thought only of those who had succeeded. There were three. The two that were transferred and the other that was frozen. The others, I didn’t think of them (1IVF; 5OE; 1ST; 1CE).

The fact that embryos are created and manipulated by health professionals outside the woman’s body, in a laboratory setting away from the prospective parents’ gaze, makes emotional detachment easier as there is no opportunity for them to become familiar or close. However, some patients undergoing IVF recognize that they are not given any space or time by the medical team to forge an emotional bond with their embryos, or even to grieve the lost/discarded embryos. Not only does this issue remain largely undiscussed in the clinical encounters, it is common for health professionals to use a metaphorical discourse that emphasizes the functional view of the process. For this purpose, they use specific vocabulary, such as ‘to develop, to select, to transfer, to freeze’, which refers to a mechanical and utilitarian approach instead of an emotional one. Some of the professionals even seek to withdraw any humanness from the embryos, thus referring to them in statistical terms, namely as possibilities of the woman getting pregnant or probabilities of success of treatment. This raises the question of how the utilitarian approach of both medical language and patients’ metaphors intersect:

Rita: [S]he [the doctor] said: ‘You have three embryos. There are three very good embryos. One is wonderful, the other is very good and the third is … what a pity … it will remain, we will have to freeze it, but we will go for two’. ‘Two, but why? What if something is wrong with me and my body rejects the embryos?’ And she said, ‘No, no, this is not how it works, we can’t know how your body will react, but there’s nothing to indicate clinically that your body will react badly. On the contrary, your uterus is perfect, your endometrium is perfect, it turns out that the probabilities are the following’. Again, probabilities. […] ‘If we put in one it’s fifty percent, if we put in two it’s seventy percent, and the chance of twins is thirty.’ So, it was like … baker’s math. Everything super professional, I don’t know … there was no time to emotionally digest anything, nor time… it was like it was a no brainer, it was like 70, 30, 50, like, there’s no more to it than [this]… (1IVF; 3OE; 1ST; 1CE).

Sandra: And I remember, when the doctor told me … I think at the time we had seven or eight embryos. And she told me, ‘Look, these are not seven children. These are embryos, they are possibilities’. […] When I started with infertility treatments, the embryos, how can I say it, were almost seen as if… generating many embryos was almost seen as a means to an end. […] And, deep down, I think … I don’t know, it’s really quite different the way I saw things at that time, I feel that. So, that was very clear to us. We let that cycle go by, we cried, we thought ‘OK, maybe I’ll never make it’, super pessimistic. […] I felt like a failure. It didn’t feel like a loss, as we probably think about it today, or how I look at pregnancy loss (2ICSI; 9OE; 1ST; 0CE; 7DE).

As part of the biomedicalization of reproduction, embryos have a strictly material role and have a utilitarian value according to an instrumental approach: they are a valuable resource that is necessary for the fulfilment of a plan (i.e. the achievement of pregnancy). Embryos are perceived as adjuvants in several technical–medical actions that take place in a functionally prepared environment – in scientific and technological terms – to satisfy the aspiration of patients undergoing ART for biological descent. There is a metaphorical understanding of the embryo as a living being, resulting from the combination of the genetic material of at least one of the prospective parents, but not yet as human life, nor as a potential person or a future child within a parental project.

### Embryos as utilities

Embryos can be metaphorically conceptualized as utilities, as biological matter or hybrid objects that fall into the category of property, capable of being the locus of ownership and dispositional control. IVF embryos are perceived as research or reproductive material, and these different conceptions impact on decisions made about them, such as to create, store, discard or donate them; use them in research; or place them in a uterus. The utility of embryos shifts within different stages of each parental project and can alter the type of relationship established with different types of embryos. Embryos that do not develop or get implanted may be merely seen as the failure of a given reproductive technique:

Eva: All those that were healthy were used. […] They [the doctors] always implanted all those that were healthy. Once it was just two and the second time it was … it was just one. […] It is like this: as it was not something that was going to be used, no … That’s it. Then it depends a little on how you feel. ‘They were already my children, I had I don’t-know-how-many children there’. I didn’t… I didn’t… No … I don’t feel that […] it was… it was my children… that were wasted. I didn’t feel that way. Hmm… It was just, really, at the time, after we knew that those were healthy and knowing that I was pregnant… […] those… the ones that had the pathology were the failed result of… fertilization. So, no … […] The goal was really just the implantation of… of the healthy ones. That was the goal (2IVF; 3EO; 0ST; 0CE).

In contrast, upon successful implantation, the utility of embryos can be converted, and ‘healthy embryos’ may become surplus. Some patients undergoing IVF perceived themselves as having decisional authority about the fate of their surplus cryopreserved embryos, which can be thawed and discarded, according to the utility principle. Beneficiaries have the contractual right to decide to dispose of those spare frozen embryos when they lose their usefulness for the purpose previously established (i.e. for the woman to get pregnant and the prospective parent/s to have biological children):

Sandra: I started using, at the time, I used the expression of liberation, it is the liberation of my embryos. It is not elimination, nor destruction, that’s even worse, because it seems that I have life there and I am destroying them. So, it’s a bit ‘OK, you are frozen here, you are here on stand-by…’. It’s a bit like looking at it as a mission, isn’t it? It doesn’t make sense to stay frozen… yeah, I was trying to avoid the word utility here, but I have to say, as if they had already had their usefulness and so now, let’s go, let’s go release them, off you go! […] There was an emotional connection, because if I hadn’t written them a letter, if I hadn’t painted the seven embryos… I painted the seven embryos as if they were inside my womb, all at the same time (2ICSI; 9OE; 1ST; 7CE).

There is a decision in favour of destruction of the embryos, where engagement with the embryos as property prevails, although this is articulated along with an emotional involvement with the cryopreserved embryos. This interviewee says she prefers to classify the option of destroying the embryos as ‘liberation’ – as opposed to ‘elimination’ or ‘destruction’ – a term she associates with the notion of the embryo as a source of life. Her description of this liberation could be said to reflect her involvement with those embryos as like waiving property rights over things that have become obsolete, that have lost ‘their usefulness’.

### Embryos as offspring

The perspectives of ART beneficiaries on IVF embryos can change over time, throughout their therapeutic trajectories – especially after the birth of a baby conceived from the same batch as the remaining cryopreserved embryos – as they confer on them a different reality (i.e. an objective materiality). The already-born IVF child becomes living and embodied proof that an embryo *in vitro* has the potential to become a person, a human being, at some point in the future. The spare embryo is no longer viewed as biological material, but as a genetically-alike sibling of their own daughter/son (genetic relatedness) and consequently an unborn child (personhood and humanness):

Rita: To think, hey, it’s an embryo, with the same genetics, practically identical to your daughter’s. It’s a sister. Suddenly, to be able to cross the barrier that I had put up on this subject, it’s a human being, and to say that … calm down, there will be someone in the world who will have, someone who is, like, my daughter’s sister, my daughter… because she is a sister, genetically speaking. (…) it’s not biological material, it’s an embryo, which has exactly the same, the same configuration as my daughter’s… it’s a feeling… that’s why, it’s because of my daughter. It’s because now that I’ve seen the baby grow, the baby was born, she is a person… she has hair of a certain colour, eyes of another, the shape of her face, she speaks this way, she is a whole person and that, that embryo is now a project similar to what my daughter was (1IVF; 3OE; 1ST; 1CE).

Some interviewees use a narrative that reinforces the personhood and humanness of the embryos created *in vitro*, although the words ‘person’, ‘human being’ or ‘human life’ are not always mentioned. For them, the embryo metaphorically represents a source of life from the beginning of fertilization, from the moment when the male and female gametes fuse, even if both members of the couple do not genetically contribute in equal terms. There is an opposition between cells (gametes are comparable with other body products that can be donated to others such as blood/platelets) and embryos (as human life and a real baby for whom prospective parents already have affection). The metaphorical categorization of the embryo as a human being or potential person, as a member of the moral community of humanity, has both symbolic and practical significance:

Laura: For me […] from the moment that the egg and the sperm come together, there is life. They are no longer cells. What the donor gave me was a cell. And that’s what I’ve been giving to other people. […] I’m a blood donor, so I also give blood to someone. I give platelets, it also gives platelets. And that person gave that cell … gave … which combined resulted in that embryo. And for me, that embryo is already my baby. It’s… it’s… He’s… I think they’re already 5 days old. It is a 5-day-old embryo. I do consider it as … For me it is already a life. For me it is a life, yes. And … and I talk to him a lot. Why? Because I think I already have affection for, for that being that is there (1IVF; 5OE; 1ST; 1CE).

Nevertheless, the genetic contribution of a third party (oocyte or sperm donor) can also be problematic and not always easy to accept. The future embryo created *in vitro* should, beneficiaries sometimes feel, be the materialization of the parental project that was idealized by the two partners (‘communality’) and therefore should also embody the equal genetic contribution of both members of the couple, thus symbolizing their union (with the value of genetic relatedness perceived as a principle of justice):

Paula: If this is a project of the two of us, I think we have to be on an equal footing, okay? […] So, since we were on an equal footing, man, it had to be from the beginning to the end. In other words, it’s either our son, or I would have to be convinced to… accept sperm from a third party. […] Because this is meant to be for both, this is a project of both, so there has to be balance, it’s either both or it is neither of us. […] I think it wouldn’t be fair for him. Because it was something he always wanted more than I did, and then I was going to be the mother, right? But he would not be the biological father afterwards. And that made me a little confused, because I didn’t think it was fair to him. Because if I wasn’t the mother, I wouldn’t be confused. But he wasn’t the father, that couldn’t happen! […] Because I was going to gestate, so it would be mine anyway (3ICSI; 5OE; 0ST; 0CE).

The use of gamete donation is not perceived the same way regardless of whether the reproductive material comes from a male or female donor. In fact, ART seems to rhetorically reinforce either the value of genetic relatedness for kinship, and parenthood in particular (the genetic contribution of at least one of the prospective parents), or the biologization of motherhood. In the case of oocyte donation, the focus is not on the genetic component but on the biological process. For the future mother, what matters may be not only the genes, but also the experience of pregnancy and childbirth. The representation of motherhood and the embodied experience of pregnancy is thus reconfigured within one’s biography. The emotional connection with the embryo and its evolution to a fetus *in utero*, during pregnancy, is articulated and overlaps with the genetic tie (genetic patrimony), preserving the understanding of the embryo as a symbol of the couple. The complex, composite and apparently contradictory nature of the judgements and meanings attributed to the embryo – which is a ‘metaphorical hybrid’ – thus derives from the binomial affective–genetic bond.

### Embryos as a counter-gift

The therapeutic use of ART involves the engagement of beneficiaries in a set of procedures and techniques in order to achieve a successful pregnancy and consequently accomplish the parental project. However, completing the family or not being able to carry out a pregnancy due to personal, medical or regulatory reasons (e.g. if the woman reaches the upper age limit for ART treatments) can lead to new ways of acting and thinking, as well as to the reshaping of meanings attributed to embryos *in vitro*.

It is in these contexts that the metaphorical figure of the embryo as a ‘gift’ or, more significantly, as a ‘counter-gift’ is likely to emerge. This metaphorical figure is understood in terms of a three-fold moral obligation supported by the relational dynamics ‘giving–receiving–reciprocating’, based on a logic of embryo donation (to other couples or to research) understood as a contribution without expectation of return or even as a form of retribution. This reconfiguration of the status–purpose binomial of these embryos – through the suspension of the parenting framework and the transition to a donation scenario – has repercussions for the bonds that patients undergoing IVF establish with these embryos over time:

Paula: [Embryos could] achieve that goal elsewhere. They would have the same status and the same objective, but elsewhere. So, you were going to give the other person the chance to … be happy. […] Even if one day I was told ‘Look, that child was born with your egg’, it would naturally be very emotional, but I doubt I would feel it was my child. It’s a bad comparison, but it’s like parents who have children they do not want and give them up for adoption, or who are taken away for adoption. After all, they are also giving other couples the opportunity to be happy, right? […] It was never mine. I mean, it was inside me, it was generated inside me but, but it no longer felt like mine. If they kept them in a refrigerator, it would make me confused, it would be as if we were wasting time. Because their place would be to make someone happy (3ICSI; 5OE; 0ST; 0CE).

The renouncement of the original parental project under which an embryo was originally created somewhat loosens the affective and biological ties eventually established with that same embryo. The preponderance of singularizing emotional or genetic ties with this entity is mitigated by the metaphorical grammar of the ‘gift’: the embryo represents an act of solidarity that transcends the utilitarian or contractual discourse, in view of the realization of other people’s parental project. In donating to another couple, the meaning and status attributed to the embryo – a ‘potential child’ – as well as the associated purpose – the realization of (another) parental project – persist, establishing a parallel with the adoption scenario.

In contrast, in the scenario where the embryo is donated for a different purpose, that of scientific research, a different oscillation occurs in the status attributed to the embryo. It is not just the purpose that changes; it is the status that, implicitly, is also reconfigured:

Paula: Given our awareness of how difficult it is, this whole process, and the mystery of this question of life and everything, they would certainly be donated to science, yes. They wouldn’t be destroyed. I mean, everything we can contribute to… yes… we’re there. […] [The embryos are then] Manipulated, altered … […] I think it’s just like after you die, whether your organs are donated to science or not, I think it’s a ‘Yes’. It’s a ‘Yes, please help someone, I don’t know, who has a disease, find the cure for all these sorrows’, isn’t it? If you can contribute with anything, that’s it, it’s not a loss, it’s a letting go, gaining another, another life (3ICSI; 5OE; 0ST; 0CE).

Making the embryo available for research – and thus an object of intervention and manipulation – presupposes the inversion of the status ‘potential child’ within a parental project. The embryo is compared with an organ, and is understood according to a functional perspective as a resource placed at the disposal of another purpose that in no way betrays its ontological, moral and social value. In fact, this distinctive purpose is extolled in the light of the collective interest of scientific progress, allowing the embryo to gain another meaning for its existence. Nevertheless, when the embryo is metaphorically conceptualized in terms of personhood, namely as a potential person – and consequently a future real baby – this understanding thus shapes the decision about the fate of cryopreserved embryos. The option to donate surplus frozen embryos to other ART beneficiaries was unacceptable for some of the interviewees given the strangeness of having the embryos becoming a real person at some point in the future:

Lia: I don’t know, it’s complicated to think about it. But also the gift of … yes, of having a child of mine in someone else’s belly, is … That’s cool for the other person, eventually that’s cool for him [the child], but … but I would prefer it to be in mine (1IVF; 2OE; 1ST; 0CE).

In contrast, their donation for clinical research (i.e. for scientific purposes) already appears to be a decision that gives the embryo meaningful social and moral value as a contribution to a bigger, worthwhile endeavour. Turning this liminal entity into a gift helps to reconstruct its meanings, thus increasing its significance. It seems to be a way of showing gratitude and repaying the contribution of science that allows individuals or couples – who cannot conceive a biological child naturally and spontaneously – to become parents. The metaphorical association with a kind of counter-gift that appears in the speeches of some of the interviewees is related to a strong feeling of gratitude towards science and medicine – specifically, reproductive technologies and the helping hand they provide.

## Discussion

Through the social integration of new forms of highly technoscientific innovation, biomedicalization has extended and reconstituted both the organization of contemporary medical practice and the way the human body in itself is perceived, studied and embedded in new individual and collective technoscientific identities (Clarke et al., 2003).

Likewise, the human embryo created in a laboratory setting, outside the woman’s body, has posed new challenges of ‘[h]ow to think it, that is, imagine it and make it real’ (Strathern, 1992: 4). Therefore, ‘the construction of the embryonic identity is a contingent, rationally undecidable and rhetorically constructed matter’ (Fox, 2000: 172). The human embryo *in vitro*, as a new technoscientific entity, is difficult to grasp. An important feature is its ambiguity. Embryos are in a grey and ambivalent area because they are part of a parental project and, at the same time, a symbol of hope in the contribution of science to the progress of medicine in general. The embryo is also a newly-created interim category, a liminal being (Turner, 1969) in transition or ‘in between’ states, where boundaries are difficult to establish objectively.

Although embryos have been represented in bioethical and legal discourse according to two main and competing perspectives (Fox, 2000) – personhood (per-persons or legal subjects) or property (commodifiable objects) – this dualistic nature is insufficient to support an in-depth analysis of the narratives of IVF beneficiaries about their embryos, given their complexity. In line with Fox’s contribution, we argue that there is a need for a new approach to second-line ART treatments such as IVF in order to forge new understandings that could inform public and institutional discussions on reproductive choices and embryo research. There is a need for a new paradigm that (re)contextualizes the embryo created in a biotechnological environment, rendering visible its dependency upon both the pregnant woman’s body and the technology by mobilizing the cyborg metaphor (Fox, 2000). Thus it might be seen as ‘a hybrid of machine and organism’ (Haraway, 1987: 1) or as ‘embody[ing] the union of science and nature’ (Franklin et al., 1999: 166).

A discursive analysis of the scientific literature available on disposal decisions offered further insight into the many facets of the social construction of IVF embryos, despite being mainly focused on the meanings attached to surplus embryos. Goedeke et al. (2017) identified different discourses: the ‘surplus embryo discourse’, where the cryopreserved embryo is potentially problematic, particularly if it is not to be used by couples; the ‘biomedical discourse’, in which the embryo is a collection of cells or seeding material; the ‘life discourse’, with embryos being referred to as human life and given a childlike persona; the ‘limbo discourse’, in which the embryo has an interim status; the ‘kinship discourse’, where the embryo is referred to as a family member; the ‘genetic blueprint and genetically dubious discourses’, where IVF embryos emerge as potentially carrying unwanted conditions from their genetic parents or as having, in general, poorer quality than those created naturally; the ‘property discourse’, with the embryo seen as individual versus public property; and the ‘personal investment discourse’, in which the embryo is described as precious and valuable.

Nachtigall et al. (2005) note how, while undergoing IVF procedures, couples are reassured by having stored surplus embryos; this is viewed as a bonus, because at this stage they do not know how many attempts will be needed to achieve pregnancy. Attitudes towards the importance of having surplus cryopreserved embryos for a subsequent IVF cycle, in case the previous one fails, seem to prevail when patients are still in treatment and do not yet know the outcome (Svanberg et al., 2001). Spare embryos seem to represent ‘security and hopefulness’ – as they are seen as additional chances of having a successful pregnancy – rather than potential children; the decision about their fate rests on ‘practical issues’ related to family planning (miscarriage, birth of a baby) and storage limits *(ibidem*). Spare embryos also reduce the woman’s physical burden if further treatment becomes necessary.

Couples’ various complex, deeply personal conceptualizations of their stored frozen embryos – as biological tissue, living entities, ‘virtual’ children, siblings of their living children, genetic or psychological ‘insurance’, or symbolic reminders of their past infertility – contributes to their ambivalence, uncertainty and difficulty in reaching a disposition decision, which is an involved and dynamic process (Nachtigall et al., 2005). Especially for those patients who view their embryos as a symbol of their relationship, the embryo disposition decision is emotionally loaded, more often than not involving feelings of grief (Provoost et al., 2012). The ambivalence and struggle between conflicting views illustrates how the emotional nature of IVF shapes the relationships established with, and the understanding of, embryos (Haimes et al., 2008).

Nevertheless, the narratives of IVF beneficiaries about all of their embryos – not only the surplus ones – deserve thorough analysis. Many factors seem to contribute to the complex meaning-making processes involved. Empirical evidence reported in earlier studies suggests that patients who believe family is biologically bounded by genetics tend to see their embryos as a genetic replica of an existing child (de Lacey, 2007, de Lacey, 2005, Laruelle and Englert, 1995, McMahon et al., 2003, Nachtigall et al., 2005, Soderstrom-Antitila et al., 2001) – as their virtual child whose development was suspended. In this scenario, feelings of ‘belonging’ and even of ‘ownership’ through genetics are emphasized (de Lacey, 2007).

Patients’ perception of the importance of genetic lineage versus education in parental bonding influences both the meaning-making processes and decisions about the fate of IVF embryos (Laruelle and Englert, 1995). Kato (2014) and Samorinha et al. (2014) show how couples have increasing confidence in donation for research as an option for their spare embryos. However, patients who perceive an embryo as having a childlike persona – that is, as being a 'virtual' child/person in cryostorage – tend not to donate embryos even if it was their initial choice because they associate this with their relinquishment for adoption (de Lacey, 2007, de Lacey, 2005). Likewise, some of our interviewees express some doubts and reservations about donating their spare embryos to other couples, although recognizing it ideally as a gift transaction. Paradoxically, they may authorize an option that can result, directly or indirectly, in their destruction (Laruelle and Englert, 1995), such as embryo donation for research purposes.

‘Saving and waiting’ can be two useful frameworks for examining the categorical ambiguities of leftover IVF embryos and how they are subject to different practices that aim to transform them from a reproductive remainder (i.e. excess clinical waste) to a repurposed state (Cromer, 2018a, Cromer, 2018b). They may be unwanted (no longer needed for the initial aim and without present-day purpose) yet unwastable (too precious to discard). By offering redemption for patients who have spare embryos and/or by recognizing their perceived potential and value, embryos can be converted into revalued forms – either as precious pre-born children that can be adopted by other couples or as a valuable and promissory research material for scientific advances – thus shaping their new future uses or identities, even if deferred in time (*ibidem*).

Patients who choose to donate their frozen embryos to other couples and position themselves at greater emotional distance from them tend to view the family as a relational unit, where the nurturing role is emphasized over the genetic connection, which is undeniable but reduced to a mere biological fact (de Lacey, 2007). Genetics can sustain different perspectives – from that of kinship to that of embryos seen as goods delivered for a service – suggesting that the meaning given to the genetic factor varies according to context (i.e. an individual’s therapeutic trajectory) rather than inherently implying kinship (Riggs, 2018). Embryo donation is seen as the donation of reproductive seeding material – tissue or inanimate cells that have the potential to become a child – and represents giving those embryos an opportunity for life, drawing attention to the emotional attachment emerging from the embodied experience of pregnancy (de Lacey, 2007). Some authors discuss the need to differentiate between parents and genitors (Laruelle and Englert, 1995). Other studies have shown that patients’ main reason for donating spare embryos is a desire to help other infertile couples fulfil their family-building goals because they have themselves received help; still, compared with oocyte donors – who mainly consider that they are only giving away a cell – embryo donors are more likely to think of their embryos as their potential children (Soderstrom-Antitila et al., 2001). Moreover, in the case of the USA, both racial constructs and religious convictions sometimes shape selective decision-making within embryo adoption programmes (Cromer, 2020). In contrast, as donor couples cannot choose the receivers of their spare embryos in Portugal, factors such as race and religion do not play a role in embryo donation.

As described by many researchers, the emotional and technological ambivalence of the personal and embodied experience of IVF (often associated with contradictory feelings, albeit becoming normalized as a way of life) affect women’s lives, self-identities and bodies (Franklin, 2013). By enabling women to experience, either physically or emotionally, implantation and pregnancies that end in miscarriages, ‘IVF ironically intensifies the very deficit it is intended to mitigate’ (Franklin, 2013: 218), paradoxically producing an unexpected and opposite outcome for which it is impossible to be prepared, as it removes the possibility of closing the struggle with infertility.

Early miscarriage, besides representing ‘the loss of possibilities’, also constitutes a unique form of loss and a special type of bereavement because of the many ambiguities around it (when the moment of death actually occurred, what was lost and what has taken place) (Frost et al., 2007). This is due to the fact that it is an ‘imperfectly scientised’ form of death where medical knowledge does not always provide a rational causal explanation, and also for the sequestration of death in terms of privatization of suffering and grieving, thus contributing to the silence, uncertainty and isolation around this life event (*ibidem*).

In order to take account of the inevitable losses, complex decisions and individual needs within the process of reproduction, we must see the full spectrum of pregnancy outcomes, and understand the embryo as a functional living organism that exists in time with its own liminal boundaries and unfolding potentialities within vital matter, but without necessarily relying on anthropomorphized definitions (i.e. as an already-human being) (DiCaglio, 2017).

In view of the above, the discussion of the embryo framed in terms of metaphors must be connected into the larger discourse about reproductive loss, especially in early stages, and psychomedical follow-up and effective help, support or counselling.

The status of IVF embryos is thus complex and difficult to establish, describe and analyse. This status changes depending on many factors – such as whether or not the embryo is included in a parental project, and whether or not the embryo has genetic ties with the IVF couple – and embryo donation adds complexity to this analysis.

Furthermore, it is also necessary to consider the impact of the broader sociocultural context in the status of human embryos, especially the regulatory context in which beneficiaries produce meanings and take decisions concerning their embryos. The ethical-legal framework around the social uses of embryos in Portugal, and the discussions and controversies in the public space that precede and involve its evolution, are primarily characterized by focusing on the progressive extension of access to ART in the context of fulfilling parental projects. The original regulatory law, of 2006, indeed provides and regulates two different categories of human embryos: (i) embryos inserted in a parental project; and (ii) unused embryos dissociated from a parental project, donated to scientific research. However, the revisions and expansions of this ethical-legal framework are eminently directed to the extension of the right to access medical assisted procreation (MAP) techniques. This is the case, in particular, of the 2016 revision, which broadens the scope of the beneficiaries for medically assisted procreation techniques, namely its use not just for heterosexual couples (married or in similar conditions for 2 years), but for all women regardless of infertility diagnosis, marital status and sexual orientation, thus encompassing single women and same-sex female couples (Law 17/2016). Also, in 2019, the regime of confidentiality in MAP techniques was redefined, namely determining that people born through gamete or embryo donation can obtain information of a genetic nature that concerns them, as well as, provided they are aged ≥18 years, obtain information on the donor's civil identification (i.e. the donor’s full name) (Law 48/2019). Additionally, in 2020, two bills on gestational surrogacy and post-mortem insemination were also approved. However, both laws were still being regulated by the time this article was written.

This current legal and ethical framework reflects precisely the confluence of positions of several stakeholders (such as associations/representatives of groups of patients or professionals, government agencies, legislative bodies, religious associations or other organizations), being these expansions in the legal regulation the result of dynamics of revindication and criticism around ethical controversies raised, expressing different moral principles. In particular, the regulatory and cultural context of Portuguese ART reflects a specific element that can be highlighted – the still-incipient stage of embryo research, with only one research project approved by the regulatory authority for ART practice in Portugal to date. This context can benefit that the fertility clinic is still primarily appropriated – both by couples and professionals – as a space for clinical treatment of infertility (i.e. a place to have a baby), relegating conceptions as places (also) for scientific research.

A context of incipient development of scientific research restrains precisely the outbreak of public controversies surrounding the use of the embryo in the context of research in the Portuguese public space, as its donation for this purpose turns out to be (still) a predominantly inconsequential route. A more effective and profuse investigation with embryos (and increased public awareness with the publicity of research results) can favour, in turn, an intensification of public controversies and a consequent confrontation of public discourses around the status and social uses of the embryo in the context of scientific research. Moreover, as international reports on reproductive policies and practices show (IFFS, 2019), human pre-implantation embryo research remains a practice in which only a small minority of countries are actively involved. A significant proportion of countries currently have scientific experimentation on embryos legalized, and there is growth in the number of countries undertaking research projects, but data on the frequency of effective experimentations performed show a still-incipient stage of development.

This aspect of the Portuguese cultural and regulatory context can therefore constitute a fundamental difference of the interviewees’ experience, in contrast to countries where the practice of direct research with embryos is at a stage of higher development (Haimes et al., 2008). The growth of research relating to embryos in this country may provide beneficiaries with different experiences in fertility clinics, with possible impacts on their conceptions and decisions around the embryo, namely, more regular contact with medical discourses that convey less overlap between an embryo and a potential child or promise (either for the progenitors’ parental project or for another couple, in the case of donation), and are more intensively open to the appropriation of the embryo as valuable biological material for research. The increase in embryonic stem cell research can, therefore, enhance the fluidity of meanings of the embryo, reinforcing, in the spectrum of metaphorical representations produced, perspectives around it as potential research material in the doctor–patient interactions that take place in the clinical space.

## Conclusion

While there is dominant public discourse about the topic of infertility and associated treatments, conveyed not only by health professionals and policy makers, but also by myriad written publications – ranging from scientific texts (articles, medical textbooks, etc.) to popular literature (women’s magazines, self-help books, etc.) – public discourse on the IVF embryo, in contrast, remains almost limited to ethical and juridical debates that are not easily accessible to laypeople. There thus prevails a variability and complexity among beneficiaries’ utterances and the metaphorical devices they use to describe feelings towards, and meanings attached to, embryos created *in vitro*.

The public sphere constitutes an arena opposing public discourses, grounded on different moral principles which guide the arguments expressed by different political actors implicated in a dispute – that is, an issue turned into a public controversy, involving competing discourses supported by different grammars to build commonality (Lamont and Thévenot, 2000, Ylä-Antilla and Luhtakallio, 2016). These grammars – rules for expressing arguments in public rooted in Western historical and cultural contexts and mobilized by actors in modern societies as publicly available evaluation repertoires – allow constructing agreements. Furthermore, the public discourses take on different weights, as well as composite forms (different combinations), according to each societal context and according to the issues that are the subject of dispute, displaying traits of its cultural and political background (*ibidem*).

In the specific case of public discourses around the embryo’s status, no publicly sedimented and institutionalized discourses are available to describe the relationship with an IVF embryo in terms of what is socially accepted or expected. Embryo meanings and uses in the context of ART and research remain mainly in the more restricted expert sphere of academic and legal debates (debates that are not morally neutral, but associated with moral principles), and as such, not yet the object of a consolidated appropriation by lay actors as publicly available discourses, but supported by more plural and more intimate meanings.

The status, meanings and uses of the human embryo thus circulate through time and space, as it bounces between the metaphorical roles of care-receiver and care-giver. The biomedical discourse, typically objectifying, is often absorbed and reinterpreted by ART beneficiaries. Nevertheless, they may face additional doubts and dilemmas about an embryo’s fate or status depending on their moral values and sociocultural norms, sometimes expressing discomfort at the most intimate and personal level (e.g. developing feelings of loss or emotional attachment to spare embryos). While we have proposed four categories of metaphor, the social construction of an IVF embryo is complex. These embryos emerge as unstable entities, with different and sometimes contrasting metaphorical meanings coexisting in the subjective construction of each personal experience of IVF.

## Limitations

The main limitation of this study is the lack of sexual, socio-economic, cultural and ethnic diversity among participants, thus limiting its ability to capture possible differences in attitudes, preferences and views according to those variables. However, as a qualitative study, its findings are not generalizable to populations beyond the study participants in any case. To date, this study has focused on patients’ perspectives, although we intend to include professionals’ perspectives at a later stage of data collection.

## Practice implications

Despite the above-mentioned limitations, this research offers preliminary insights into conceptualizations of the embryo *in vitro* through metaphor-based reasoning, as well as exploring the type of arrangements that patients undergoing IVF develop and negotiate with themselves and with health professionals. This study has produced a set of findings that contribute to our understanding of the complexity and changeability of meaning-making about embryos, providing avenues for future research. In fact, little has been published on lay conceptions of human embryos created in a laboratory setting – except insofar as they relate to surplus frozen ones. Studies have focused on fertility patients who currently have spare embryos stored and their preferences for disposition, given the challenges that this entails for both clinicians and policymakers. Our findings thus reflect beneficiaries’ views of the statuses and possible fates of IVF embryos at each stage of treatment, whether or not they discuss these with medical professionals or counsellors. Sometimes, the situations faced by IVF users are emotionally unbearable to them, and sometimes, other possibilities that would be more morally acceptable are not available, thus creating an additional strain. Although this study has identified the four types of metaphor used by patients undergoing IVF regarding their embryos *in vitro*, it has not addressed their effectiveness in helping individuals experiencing difficult situations such as embryo loss to cope, adapt and heal. Future research should consider the impact of these metaphors either in increasing negative thoughts and feelings, such as anxiety, grief and regret, or mitigating them by enhancing well-being. Future studies should look more closely into all these embryo-specific concerns, tensions, dilemmas and uncertainties expressed by patients undergoing IVF so that their voices may inform the entire process of clinical care and public policy in assisted reproduction, not just that of embryo disposition. This metaphorical framework can be used as a visual tool to raise clinicians’ awareness of important dimensions of decision-making by ART beneficiaries about their embryos, and may also give insight about how to counsel people facing unexpected, inexplicable and disturbing events, such as early pregnancy loss. Guidelines to address this gap may involve: (i) practitioners’ improved sensitivity and deeper attention to informed consent and doctor–patient communication processes, as well as educational and counselling protocols for patients undergoing IVF/ICSI procedures, namely early and detailed disclosure – prior to treatment commencing – about all facts regarding human embryos, from their initial creation and selection criteria to their future implantation, loss, destruction or donation; and (ii) a periodic follow-up, involving discussions with patients about their reproductive goals and values as well as forms of moral reasoning that are particular to each case and may evolve with time and context (i.e. that can change depending on the circumstances and the patients’ personal experience at a given time). This study has important implications for quality clinical practice (patient-centred care), suggesting that there is a need for more psychosocial support and counselling prior to, during and after IVF/ICSI treatments. In fact, the European Society of Human Reproduction and Embryology has published some recommendations for the optimal management of routine psychosocial care at infertility and ART clinics (Gameiro et al., 2015), but these do not address the specific psychosocial needs that patients experience across their treatment pathway in relation to their IVF embryos.

## Declaration

The author reports no financial or commercial conflicts of interest.
