# Supplementary material for: In-vitro metaphors: ART beneficiaries’ meaning-making about human embryos in the context of IVF in Portugal
Source: Reprod Biomed Soc Online. 2021 Jun 29;13:62–74. doi: 10.1016/j.rbms.2021.05.003 (PMC8322126; doi:10.1016/j.rbms.2021.05.003)
Supplement: Supplementary data 1 [file mmc1.docx]

**Interviewees' therapeutic trajectory**

| Interviewee No. | Interviewee pseudonym | Type of infertility treatment (IVF, IA, ICSI) | No. of treatment cycles | Number of IVF embryos obtained/fertilized eggs | Transferred embryos | Non-viable embryos (non-transferable/undeveloped) | Surplus cryopreserved embryos (at the date of the interview): Y/N and *n* | Treatment stage (at the time of the interview): finished, in process, etc. |
| --- | --- | --- | --- | --- | --- | --- | --- | --- |
| F1 | Alice | IA | 1 | n/a | n/a | n/a | n/a | Treatment ended (child born from spontaneous pregnancy) |
| F2 | Clara | IA | 1 | n/a | n/a | n/a | n/a | Treatment ended (pregnancy obtained – twins); third child born from spontaneous pregnancy |
| F3 | Eva | IVF | 2 | 2 | 2 | 0 | 0 | Treatment ended (no pregnancy obtained) |
|  |  |  |  | 1 | 1 | 0 |  |  |
| F4 | Miranda | ICSI | 2 | 2 | 2 | 0 | 0 | Treatment ended (pregnancy obtained –twins) |
|  |  |  |  | 2 | 2 | 0 |  |  |
| F5 | Sara | IVF | 1 | 12 | 4 | 0 | 0 | Treatment ended (pregnancy obtained) |
| F6 | Brenda | IVF | 3 | 8 | 1 | 7 | 0 | Treatment ended (pregnancy obtained) |
|  |  |  |  | 16 | 2 | 13 |  |  |
|  |  |  |  | n/a^a^ | 1 | n/a^a^ |  |  |
|  |  |  |  | 3 | 2 | 0 |  |  |
|  |  |  |  | n/a^a^ | 1 | n/a^a^ |  |  |
| F7 | Clarice | IA, IVF | 1 (IA) | n/a | n/a | n/a | n/a | Treatment ended (pregnancy obtained) |
|  |  |  | 1 (IVF) | 8 | 2 | 3 | 0 |  |
|  |  |  |  | n/a | 3 |  |  |  |
| F8 | Irene | IVF | 2 | 12 | 0 | 12 | 1 | Treatment ended (pregnancy obtained); considering transferring the cryopreserved embryo |
|  |  |  |  | 11 | 1 | 5 |  |  |
|  |  |  |  | n/a^a^ | 1 | n/a^a^ |  |  |
|  |  |  |  | n/a^a^ | 1 | n/a^a^ |  |  |
|  |  |  |  | n/a^a^ | 2 | n/a^a^ |  |  |
| F9 | Ismael | IVF | 3 | 2 | 1 | 1 | 0 | Treatment ended (pregnancy obtained – twins) |
|  |  |  |  | 1 | 1 | 0 |  |  |
|  |  |  |  | 3 | 2 | 1 |  |  |
| F10 | Olivia | IA, IVF | 2 (IA) | n/a | n/a | n/a | 0 | Pregnancy not obtained; treatment suspended and expecting to have a new cycle in the coming months |
|  |  |  | 2 (IVF) | n/a | n/a | n/a |  |  |
|  |  |  |  | 3 | 1 | 2 |  |  |
| F11 | Selma e Gabriel | IVF | 2 | 8 | 2 | 2 | 0 | Treatment ended (pregnancy obtained in the last treatment); second child born from spontaneous pregnancy |
|  |  |  |  | n/a^a^ | 2 | n/a^a^ |  |  |
|  |  |  |  | n/a^a^ | 2 | n/a^a^ |  |  |
|  |  |  |  | 8 | 2 | 0 |  |  |
|  |  |  |  | n/a^a^ | 2 | n/a^a^ |  |  |
|  |  |  |  | n/a^a^ | 2 | n/a^a^ |  |  |
|  |  |  |  | n/a^a^ | 2 | n/a^a^ |  |  |
| F12 | Laura | IVF (use of donated eggs) | 1 | 5 | 2 | 2 | 1 | Pregnancy obtained; transfer of cryopreserved embryo scheduled |
| F13 | Ida | IVF | 2 | 5 | 2 | 3 | 0 | Treatment ended (no pregnancy obtained) |
|  |  |  |  | 5 | 2 | 3 |  |  |
| F14 | Dora | ICSI | 1 | 5 | 2 | 3 | 0 | Treatment ended (pregnancy obtained) |
| F15 | Denise e Leonardo | IVF-ICSI (use of donated eggs in the second cycle) | 2 | 1 | 1 | 0 | 3 | Pregnancy obtained; destination of cryopreserved embryos to be decided |
|  |  |  |  | 5 | 1 | 1 |  |  |
| F16 | Rita | IVF | 1 | 3 | 2 | 0 | 1 | Pregnancy obtained; considering transferring the cryo-preserved embryo |
| F17 | Vanessa | IA, IVF | 3 (IA) | n/a | n/a | n/a | 0 | Treatment ended (pregnancy obtained – twins); subsequent spontaneous pregnancy |
|  |  |  | 1 (IVF) | 4 | 2 | 2 |  |  |
| F18 | Julia | ICSI | 1 | 6 | 2 | 4 | 0 | Treatment ended (pregnancy obtained) |
| F19 | Sandra | ICSI | 2 | 9 | 1 | 0 | 7 | Pregnancy obtained (in the second cycle); decided to destroy the surplus cryo-preserved embryos |
|  |  |  |  | n/a^a^ | 1 | n/a^a^ |  |  |
| F20 | Paula | ICSI | 3 | 0 | 0 | 0 | 0 | Treatment ended (no pregnancy obtained); recourse to adoption |
|  |  |  |  | 5 | 2 | 3 |  |  |
|  |  |  |  | 0 | 0 | 0 |  |  |
| F21 | Maria | ICSI | 2 | 4 | 2 | 2 | 0 | No pregnancy obtained; considering entering a new cycle (with oocyte donation) |
|  |  |  |  | 2 | 2 | 0 |  |  |
| F22 | Roberta | IVF | 1 | 11 | 5 | 2 | 3 | Pregnancy obtained (twins); considering donating surplus embryos |
| F23 | Lia | IVF | 1 | 2 | 2 | 0 | 0 | Pregnancy obtained; waiting to start a new treatment cycle |
| F24 | Vera | ICSI | 3 | 8 | 1 | 7 | 3 | Ongoing treatment (pregnancy not yet obtained; awaiting transfer of three frozen embryos obtained in last ICSI) |
|  |  |  |  | n/a^a^ | 1 | n/a^a^ |  |  |
|  |  |  |  | 5 | 1 | 4 |  |  |
|  |  |  |  | 7 | 0 | 4 |  |  |
| F25 | Flora | IVF-ICSI | 4 | 2 | 1 | 1 | 0 | Pregnancy not obtained; intending to undergo a new (and final) cycle |
|  |  |  |  | 0 | 0 | 0 |  |  |
|  |  |  |  | 0 | 0 | 0 |  |  |
|  |  |  |  | 4 | 3 | 1 |  |  |
| F26 | Mercedes | IA, ICSI | 1(IA) | n/a | n/a | n/a | 0 | Treatment ended (pregnancy obtained – twins); surplus embryo was donated |
|  |  |  | 1(ICSI) | 3 | 2 | 0 |  |  |
| F27 | Olivia | IVF-ICSI | 2 | 12 | 2 | 2 | 2 | Ongoing treatment (awaiting possible transfer of two cryopreserved embryos); no pregnancies yet obtained |
|  |  |  |  | n/a^a^ | 2 | n/a^a^ |  |  |
|  |  |  |  | n/a^a^ | 2 | n/a^a^ |  |  |
|  |  |  |  | n/a^a^ | 2 | n/a^a^ |  |  |
|  |  |  |  | n/a^a^ | 2 | n/a^a^ |  |  |
|  |  |  |  | 4 | 1 | 2 |  |  |
|  |  |  |  | n/a^a^ | 1 | n/a^a^ |  |  |
|  |  |  |  | 7 | 0 | 5 |  |  |
| F28 | Barbara | IVF | 1 | 10 | 1 | 6 | 3 | Pregnancy obtained; not decided on the three cryo-preserved embryos |
| F29 | Matilda | IVF | 3 | 0 | 0 | 0 | 2 | Pregnancies obtained in the second and third treatment cycles; not decided on the two cryo-preserved embryos |
|  |  |  |  | 5 | 1 | 1 |  |  |
|  |  |  |  | n/a^a^ | 1 | n/a^a^ |  |  |
| F30 | Amanda | IVF | 1 | 18 | 2 | 10 | ^6^ | Pregnancy not obtained; awaiting transfer of the remaining six cryopreserved embryos |

IVF, in-vitro fertilization; ICSI, intracytoplasmic sperm injection; AI, artificial insemination.

^a^Cycle without ovarian stimulation (use of cryopreserved embryos obtained from a previous cycle).
